# Utilization of Forest Residues for Cellulose Extraction from Timber Species in the High Montane Forest of Chimborazo, Ecuador

**DOI:** 10.3390/polym16192713

**Published:** 2024-09-25

**Authors:** Dennis Renato Manzano Vela, Cristina Nataly Villegas Freire, Rolando Fabian Zabala Vizuete, Ana Carola Flores Mancheno

**Affiliations:** Facultad de Recursos Naturales, Escuela Superior Politécnica de Chimborazo (ESPOCH), Riobamba 060150, Ecuador; cvillegas@espoch.edu.ec (C.N.V.F.); rzabala@espoch.edu.ec (R.F.Z.V.); acmancheno@espoch.edu.ec (A.C.F.M.)

**Keywords:** cellulose extraction, forest residues, sustainable forestry practices

## Abstract

The present study explored the extraction of cellulose from forest residues of four timber species, namely *Cedrela montana* Moritz ex Turcz, *Buddleja incana* Ruiz & Pav, *Vallea stipularis* L. f. and *Myrsine andina* (Mez) Pipoly, in the high montane forest of Chimborazo province, Ecuador, for the sustainable utilization of leaves, branches, and flowers. An alkaline extraction method was used on the residues without the need for prior degreasing. An ANOVA analysis was applied to evaluate significant differences in cellulose extraction yields among the species’ residues. The characterization techniques used were Fourier transform infrared spectroscopy (FTIR) and polarized light optical microscopy, which confirmed the successful extraction of cellulose with characteristics comparable to standard cotton cellulose and other traditional species. The results showed significant variations in cellulose yield among the species, with *Vallea stipularis* L. f achieving the highest yield of 80.83%. The crystallinity of the samples was clearly evidenced by the polarity of the light in the samples during microscopy, demonstrating that the residues can be a viable and sustainable source of cellulose, contributing to a circular economy and reducing the environmental impact of forest waste.

## 1. Introduction

The valorization of forest residues has become a fundamental paradigm in green chemistry and the circular bioeconomy, presenting significant opportunities for obtaining high value-added materials [1,2]. In this context, the extraction of cellulose from residual lignocellulosic biomass emerges as a promising strategy for the sustainable management of forest resources and the development of new materials [3,4,5].

High montane forests, ecosystems of great ecological and economic importance, are characterized by their rich biodiversity and their crucial role in water regulation and carbon capture [6]. In Ecuador, specifically in the province of Chimborazo, these forests are home to various timber species that, after being harvested, generate considerable volumes of forest residues [7,8]. In this study, forest residues from four timber species characteristic of the high montane forest of Chimborazo, Ecuador, were used, namely *Cedrela montana* Moritz ex Turcz, *Buddleja incana* Ruiz & Pav, *Vallea stipularis* L. f., and *Myrsine andina* (Mez) Pipoly. The choice of these species is based on their abundance and potential for waste generation after their exploitation in the province. According to data from the Ministry of Environment and Ecological Transition of Ecuador, it is estimated that more than 30% of the timber biomass harvested in this region generates residues that currently do not have an established commercial use, which underlines the potential of these by-products for the production of high value-added materials [9]. The efficient utilization of these by-products for cellulose extraction would not only mitigate the environmental impact associated with their disposal but also provide a renewable source of this versatile biopolymer [7].

Cellulose, as the most abundant natural polymer in the biosphere, possesses exceptional physicochemical properties that make it a material of interest for multiple industrial and technological applications [10]. Its chemical structure, based on β-D-glucopyranose units linked by β-1,4-glycosidic bonds, imparts high mechanical strength and thermal stability to cellulose, desirable characteristics in the production of advanced materials [11,12].

The efficient extraction of cellulose from forest residues presents significant technical challenges, primarily due to the complex structure of the plant cell wall and the presence of lignin and hemicelluloses [13]. In this study, an innovative alkaline extraction method has been implemented, eliminating the need for prior degreasing, thus simplifying the process and reducing the use of organic solvents [14,15]. The use of alkalis in cellular fractionation of lignocellulosic biomass may be considered expensive compared to other approaches, such as the use of acids or organic solvents; however, it offers advantages in terms of efficiency, product purity, and alignment with green chemistry principles. Alkalis, such as NaOH, are effective in breaking down complex lignocellulosic structures, facilitating the dissolution of hemicelluloses and lignin, and allowing for better recovery of high-purity cellulose [16]. Furthermore, alkaline treatment improves cellulose crystallinity, which is crucial for advanced applications, such as nanomaterial and biocomposite production [17].

Alkaline treatment is more sustainable in the long term, as alkaline solutions can be regenerated and reused in industrial processes, reducing the environmental impact and operational costs at larger scales [18]. Although there are more economical alternatives, such as enzymatic or mechanical treatments, these are usually less efficient for processing large volumes of biomass or require additional steps to achieve the same cellulose purity [19]. In this context, the selection of alkalis is justified by their ability to maximize the yield and quality of the final product. This approach not only optimizes the efficiency of the process but also aligns with the principles of green chemistry by minimizing the environmental impact and improving the sustainability of the extraction [20].

For the characterization of the extracted cellulose, advanced analytical techniques have been employed. Fourier transform infrared spectroscopy (FTIR) allows precise identification of the characteristic functional groups of cellulose and the assessment of its purity [21,22,23]. Complementarily, polarized light optical microscopy offers a detailed view of the morphology and crystallinity of the cellulose fibers, providing crucial information about the structure and properties of the obtained material [24,25,26].

Despite advancements in the field, there is a knowledge gap regarding the characterization and utilization of specific timber species from the high montane forest of Ecuador for cellulose extraction [6]. The variability in the chemical composition and anatomical structure of these species can significantly influence the efficiency of the extraction process and the properties of the obtained cellulosic material [27,28,29].

In this context, the main objective of the present study is to evaluate the potential of forest residues from selected timber species in the high montane forest of Chimborazo, Ecuador, for the extraction of high-purity cellulose using an optimized alkaline method. This method aims to uncover specific characteristics of these species under appropriate extraction conditions, with an emphasis on process sustainability and the quality of the final product.

This study is expected to serve as a starting point for future research on the application of cellulose extracted from forest residues in the development of high-value semi-synthetic materials, aligning with the principles of the circular bioeconomy and green chemistry, thus contributing to the sustainable management of natural resources in Ecuador.

## 2. Materials and Methods

### 2.1. Study Area and Sample Collection

The research was conducted in two montane forests in the province of Chimborazo, Ecuador, namely the Leonán de Llucud forest and the Cochapamba forest. The Leonán de Llucud forest, belonging to the San Pedro de Llucud Association in the Chambo canton, is situated at an altitude between 3200 and 3600 m above sea level. Similarly, the Cochapamba forest, located in the Penipe canton, shares similar altitudinal and climatic characteristics, exhibiting a rugged geomorphology characteristic of the Andean zone of Ecuador.

### 2.2. Sample Collection and Taxonomic Identification

A systematic and bioethical approach was implemented for sample collection. The methodology included stratified sampling, selecting representative areas within the forests to ensure broad coverage of plant diversity. Bioethical practices were applied to minimize ecosystem disturbance, and the necessary permits were obtained from the relevant authorities. Each collected specimen was meticulously labeled with georeferenced data, the collection date, and collector information, thereby ensuring traceability and data integrity. The collection process was approved by the Bioethics Committee of the Escuela Superior Politécnica de Chimborazo.

### 2.3. Herbarium of Dendrological Samples

The collected samples were subjected to a herbarium process following standardized protocols. This process included controlled drying at 65 °C and pressing of the specimens, ensuring optimal preservation of the essential morphological characteristics for subsequent taxonomic analysis.

### 2.4. Taxonomic Identification

The taxonomic identification was carried out through a comparative morphological analysis. Specialized dichotomous keys, updated taxonomic literature, and comparisons with reference specimens were used. The final verification was conducted at the QCA Herbarium of the Pontificia Universidad Católica del Ecuador, employing stereoscopic microscopy techniques and consulting with experts in Andean plant taxonomy. The identified and confirmed species were *Cedrela montana* Moritz ex Turcz, *Buddleja incana* Ruiz & Pav, *Vallea stipularis* L. f., and *Myrsine andina* (Mez) Pipoly.

### 2.5. Cellulose Extraction

Prior to the cellulose extraction process, the fat–wax fraction was measured in the four species studied. A total of 50 g of dry samples of plant residues (leaves, flowers, and branches) from each species, previously ground to a uniform particle size, were taken. The samples were placed in Soxhlet extraction cartridges and exposed to a continuous extraction cycle with hexane as the solvent for 8 h. Once the extraction was completed, the hexane was recovered by distillation, leaving the fat–wax fraction in the collection flask. The recovered fat–wax fraction was weighed after removing any trace of residual solvent by evaporation under reduced pressure. The mass of the fat–wax fraction was calculated based on the initial amount of dry plant material, obtaining results below 4% by weight for all species. These results indicated that the fat–wax fraction present in the biomass samples is minimal and does not require additional treatment for its removal before the alkaline cellulose extraction process. The small amount of fat–wax was further reduced during the alkaline treatment, which justified the elimination of prior degreasing, thus simplifying the process and reducing the use of organic solvents. The residual amounts of fat–wax are low enough not to interfere with the quality of the extracted cellulose, as evidenced in subsequent purity and characterization analyses [14]. To continue with the protocol, 50 g of plant residues (leaves, flowers, and branches) were used in each repetition (three in total) for each species. The extraction began with the preparation of a 10% (*w*/*v*) NaOH solution. The residue particles, previously reduced to a uniform size through cryogenic grinding, were immersed in this solution. The mixture underwent a cooking process at 90 °C for 10 min, followed by controlled cooling to room temperature for 20 min. Subsequently, successive washes with distilled water were performed until a neutral pH was reached, as verified using a calibrated pH meter. Then, the material was dried at 65 °C in a forced convection oven until a constant weight was achieved.

The next stage involved a treatment with 4% (*v*/*v*) sulfuric acid at boiling temperature for one hour, followed by washes until a neutral pH was reached. A treatment with 3.5% (*w*/*v*) sodium chlorite was applied for 3 h, including a heating phase in a water bath at 95 °C for 40 min. After additional washes, a treatment with 20% (*w*/*v*) NaOH was performed under constant agitation at 200 rpm for one hour. The process concluded with a final treatment of 0.5% (*w*/*v*) sodium chlorite for one hour, followed by thorough washing and drying at 65 °C until a constant weight was achieved.

### 2.6. Characterization Techniques

#### 2.6.1. Fourier Transform Infrared Spectroscopy (FTIR)

FTIR analysis was performed using an ORIGIN JASCO FT/IR-4100 Type A spectrometer: JASCO Corporation, Tokyo, Japan. The spectra were obtained in the range of 4000 to 550 cm^−1^, with a resolution of 4 cm^−1^ and 64 scans per sample. The samples were prepared using the KBr pellet technique. The resulting spectra were compared with a high-purity (>99%) cotton cellulose standard to identify the characteristic cellulose bands and to evaluate the effectiveness of the extraction process.

#### 2.6.2. Polarized Light Optical Microscopy

Morphological and crystallinity analysis was performed using a Zeiss Axio Imager.M2m microscope: Carl Zeiss AG, Oberkochen, Germany equipped with polarization filters. The samples were prepared in diluted aqueous suspensions and observed at different magnifications (10×, 40×, and 100×) to examine the fibrillar structure and the characteristic birefringence of crystalline cellulose.

### 2.7. Data Analysis

The FTIR spectral data were analyzed for baseline correction and normalization. Microscopy images were analyzed using ImageJ software: Version 1.53t to quantify the morphological parameters of the fibers. Statistical analysis was performed using R Studio: Version 2023.06.1 (Build 524), including analysis of variance (ANOVA) and Tukey’s post hoc tests to evaluate significant differences in cellulose extraction yields according to TAPPI 205 standards among the different species [30].

## 3. Results

### 3.1. Mass Fractions of the Components in Each Species

Table 1 presents the cellulose fractions in the forest residues of the studied species, with a hemicellulose content of 26.4 ± 1.3%, determined by hydrolysis, while the lignin content was 20.1 ± 1.8%, evaluated using the Klason method. The ash content was 2.1 ± 0.3%, obtained by calcination at 550 °C, and the fat and wax fraction extracted by Soxhlet with hexane represented 3.3 ± 0.7% of the dry weight of the samples. These values indicate a relatively homogeneous composition in terms of the structural components of the analyzed species, with minimal variations between them.

The average molecular weight of cellulose measured by viscometry was 463,281.3 ± 10,549.6 g/mol, with a degree of polymerization (DP) of 2849.35 ± 65.0, which is in accordance with the values reported in the literature for eucalyptus kraft pulps, ranging between 2100 and 4750. This high degree of polymerization suggests greater structural integrity of the polysaccharides, especially glucose, which could influence the resistance of the fibers to degradation processes, such as enzymatic or chemical hydrolysis. The relatively high concentration of hemicellulose indicates a high presence of xylans, arabinose, galactose, and mannose, which are common in plant cell walls.

### 3.2. Amount of Extracted Cellulose

#### 3.2.1. Cellulose Fiber and Pulp Yield

The values refer to the percentage of cellulose fiber obtained and recovered after extraction with NaOH as well as the percentage of cellulose recovered after bleaching, considering the total percentage of cellulose in the sample, are presented in Table 2 [30]. Meanwhile, in the species *Cedrela montana* Moritz ex Turcz, a decrease in the mass fraction of cellulose is observed when comparing the values of bleached pulp obtained after delignification with the recovered fiber, especially in relation to the other species studied [31]. This phenomenon is related to the quality and intrinsic strength of cellulose fibers, which in the case of *Cedrela montana* Moritz ex Turcz seem to be composed of a higher proportion of shorter cellulose chains or with structural imperfections. These characteristics make the fibers more susceptible to degradation during the bleaching and pH adjustment stages [32]. The degradation is more pronounced in fibers that already have inherent weaknesses, resulting in an additional loss of cellulosic mass during the purification process but at the same time ensuring that the obtained product contains high-quality cellulose.

$$Cellulose\;Fiber=\frac{x}{p}\times 100$$

*x* = Amount of fiber (g).
*p* = Amount of plant material (g).

$$Cellulose\;Yield=\frac{C}{Z}\times 100$$

*C* = Amount of pulp (g).
*Z* = Cellulose fiber (g).

**Table 2:** Cellulose fiber and pulp yield.

Species	Sample Amount (g)	Fiber Amount (g)	Cellulose Fiber (%)	Pulp Amount (g)	Bleached Cellulose Pulp (%)	Total Cellulose Yield (%)
***Cedrela montana* Moritz ex Turcz**	50	32.1	64.2%	20.21	62.96%	40.41%
***Buddleja incana*** Ruiz & Pav	50	29.98	59.96%	21.12	70.45%	42.22%
***Vallea stipularis*** L. f.	50	33.12	66.24%	26.77	80.83%	53.54%
***Myrsine andina*** (Mez) Pipoly	50	30.56	61.12%	19.77	64.69%	39.43%

#### 3.2.2. ANOVA Results Cellulose Fiber and Pulp

Table 3 shows the results of a one-way ANOVA. The *p*-value (0.342) for the percentage of fiber showed that there are no significant differences between the species. In contrast, the percentage of pulp presented highly significant differences (F3,8 = 22.64, *p* = 2.9 × 10^−4^).

The Fisher LSD post hoc test is shown in Table 4, where significant differences were identified between *Vallea stipularis* L. f. and the other three species. However, no significant differences were found between *Myrsine andina* (Mez) Pipoly and *Cedrela montana* Moritz ex Turcz at 95% confidence (LSD = 5.52). When increasing the confidence level to 99% (LSD = 8.04), *Buddleja incana* Ruiz & Pav was added to this group, indicating that there are no significant differences between *Buddleja incana* Ruiz & Pav, *Myrsine andina* (Mez) Pipoly, and *Cedrela montana* Moritz ex Turcz.

For the verification of assumptions, the results in Table 5 revealed that both the residuals of the fiber percentage and the pulp percentage met the necessary assumptions to validate the ANOVA results.

### 3.3. Characterization of Extracted Cellulose

#### 3.3.1. FTIR Analysis of *Cedrela montana* Moritz ex Turcz

**Figure 1:**
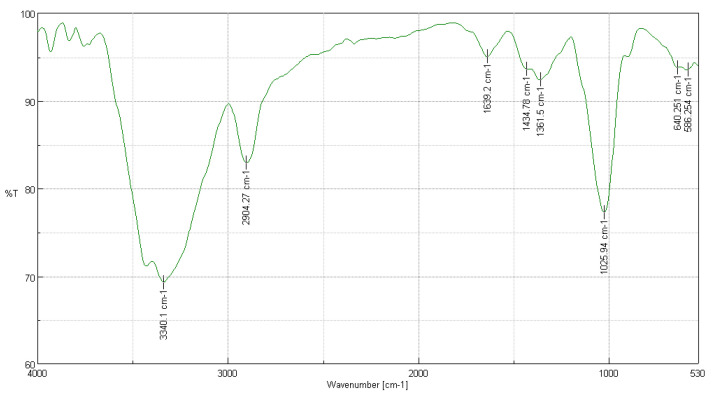
FTIR analysis of *Cedrela montana* Moritz ex Turcz.

In Figure 1, the FTIR results of the cellulose extracted from *Cedrela montana* Moritz ex Turcz are presented. The characteristic peaks observed in the spectrum were compared with a cotton cellulose sample to verify the similarity in cellulose structure [22].

The peak at 3336 cm^−1^ indicates the presence of hydroxyl groups (-OH), typical in cellulose structures, showing a transmission of 70%, similar to the 69.2824% observed in the cotton sample. This suggests a well-preserved cellulose structure. The peak at 2918 cm^−1^ (assigned to 2904.27 cm^−1^ in our sample) with a transmission of 85%, compared to 82.9496% in cotton, corresponds to the C-H stretching of methyl and methylene groups, confirming the structural similarity to cotton cellulose in terms of basic carbon–hydrogen components [33].

At 1639 cm^−1^, the observed transmission is 95%, aligning with the 95.0238% of cotton, indicating the absorption of bound water, a common phenomenon in cellulose samples. The peak at 1434 cm^−1^ reflects the stretching of C-H bonds and the presence of CH_2_ groups, with a slight difference in transmission (90% compared to 93.6327% in cotton), possibly due to minor differences in processing or the cellulose source [34].

The peaks at 1372 cm^−1^ and 1056 cm^−1^ show transmissions of 88% and 75%, respectively, comparable to the values of 92.3891% and 77.2704% in cotton, respectively, indicative of cellulose structures with C-O-C stretching in ethers and glycosidic bonds [34]. The peaks at 667 cm^−1^ and 897 cm^−1^ with transmissions of 85%, compared to 93.8052% and 93.5504% in cotton, respectively, may reflect differences in crystallinity and molecular order, influenced by the sample treatment method [35].

#### 3.3.2. FTIR Analysis of *Buddleja incana* Ruiz & Pav

**Figure 2:**
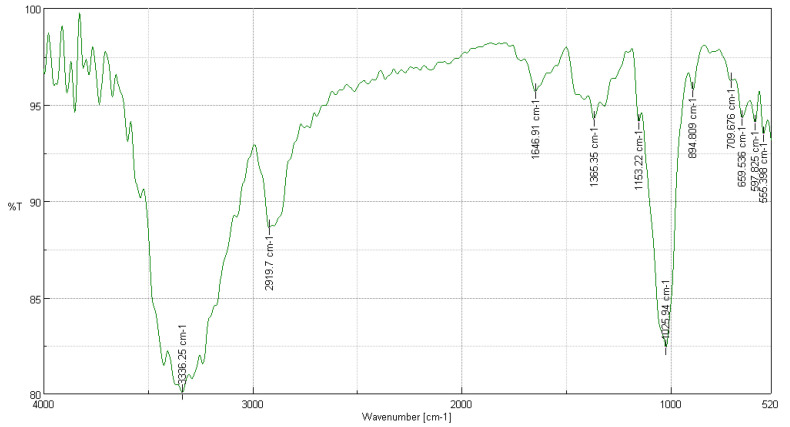
FTIR analysis of *Buddleja incana* Ruiz & Pav.

In the FTIR analysis of *Buddleja incana* Ruiz & Pav, presented in Figure 2, several significant peaks were observed compared to a cotton cellulose sample [22]. The peak at 3336 cm^−1^, indicating the presence of intramolecular hydroxyl groups, showed a transmittance of 90% compared to 80.0719% in cotton, suggesting a well-preserved cellulose structure. The peaks at 2919.7 cm^−1^ and 1646.91 cm^−1^, with transmittances of 90% and 95%, respectively, correspond to CH_2_ stretching and O-H bending due to adsorbed water, indicating similarities in the basic structures and water absorption properties. Additionally, the peak at 1365.35 cm^−1^ reflects C-H bending in the cellulose ring structure, while the peak at 1153.22 cm^−1^ suggests C-O-C bridge stretching. The C-O-C stretching in ethers and glycosidic bonds was observed at 1025.94 cm^−1^, and the beta linkage of cellulose manifested at 894.809 cm^−1^ [36]. Other peaks, such as 709.676 cm^−1^ and 659.536 cm^−1^, indicate CH2 rocking and out-of-plane OH bending, respectively, both reflecting the organization of cellulose in the fiber. Finally, the peaks at 597.825 cm^−1^ and 555.398 cm^−1^ also show specific characteristics of cellulose, with slightly lower transmittances in *Buddleja incana* Ruiz & Pav compared to cotton, which could indicate minor differences in crystallinity or molecular order within the cellulose structure [35].

#### 3.3.3. FTIR Analysis of *Vallea stipularis* L. f.

**Figure 3:**
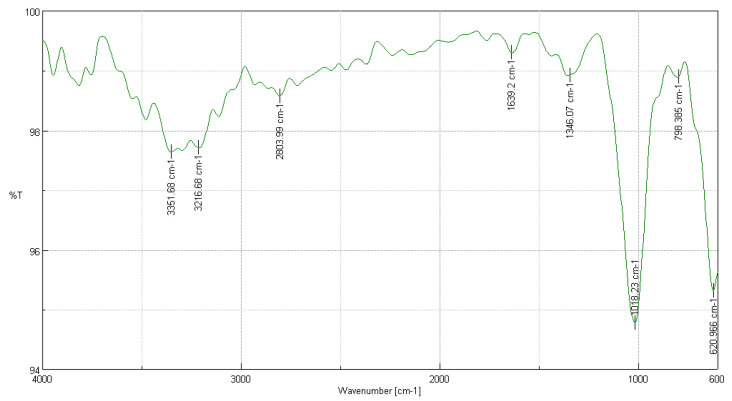
FTIR analysis of *Vallea stipularis* L. f.

In the FTIR analysis of *Vallea stipularis* L. f. shown in Figure 3, several significant peaks were observed that evidence the molecular structure of cellulose. The peak at 3351.68 cm^−1^, with a transmittance of 97.36%, is associated with hydroxyl group (-OH) stretching, indicating strong intramolecular hydrogen bonds essential for the cellulose structure. At 3216.68 cm^−1^, with a transmittance of 97.70%, intermolecular hydrogen bonds critical for the structural integrity of cellulose are observed. The peak at 2803.99 cm^−1^ reflects C-H stretching, indicating the presence of methyl and methylene groups [37]. At 1639.2 cm^−1^, a peak associated with O-H bending due to adsorbed water shows a common property in natural cellulose samples. The peak at 1346.07 cm^−1^, indicative of C-H bending, and the one at 1018.23 cm^−1^, suggesting C-O stretching and ring stretching modes, are fundamental to the cellulose structure. Additionally, at 798.385 cm^−1^, a linkage to the beta bond of cellulose is observed, and at 620.966 cm^−1^, CH_2_ rocking vibrations are present. Both reflect crucial structural components in cellulose, corroborating the high purity and proper structure of the cellulose extracted from *Vallea stipularis* L. f. [34].

#### 3.3.4. FTIR Analysis of *Myrsine andina* (Mez) Pipoly

**Figure 4:**
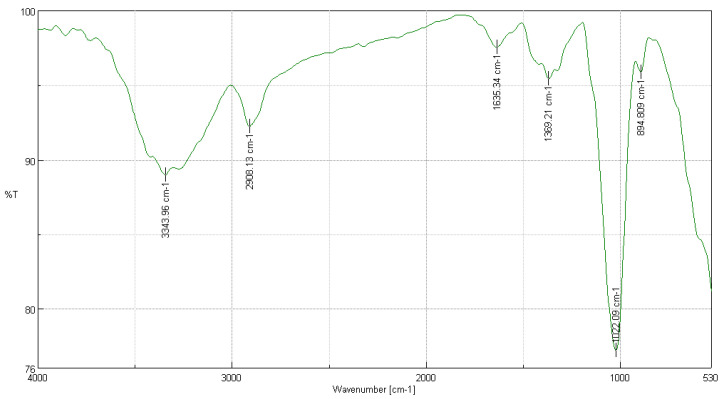
FTIR analysis of *Myrsine andina* (Mez) Pipoly.

In Figure 4, the results of the FTIR analysis of *Myrsine andina* (Mez) Pipoly are shown. Significant peaks were meticulously compared with those of a cotton cellulose sample to evaluate the quality and structure of the extracted cellulose [22]. The peak at 3343.96 cm^−1^, showing a transmittance of 88.9673%, corresponds to the stretching of hydroxyl groups (-OH), implying strong intramolecular hydrogen bonding, which is crucial for the cellulose structure. At 2908.13 cm^−1^, with a transmittance of 92.2349%, the presence of C-H stretching is observed, indicative of the basic carbon chains in cellulose [38]. The peak at 1635.34 cm^−1^ with a transmittance of 97.5537% is typical of O-H bending due to adsorbed water, reflecting a common property in natural celluloses. At 1369.21 cm^−1^, with a transmittance of 95.4629%, C-H bending is observed, which is indicative of the ring structure of cellulose. The peak at 1022.09 cm^−1^, although with a lower transmittance of 77.1867%, suggests C-O stretching and ring stretching modes, fundamental in the cellulose structure. Finally, at 894.809 cm^−1^, with a transmittance of 95.8578%, the beta linkage of cellulose is observed, indicating the typical polymeric structure of cellulose. These results demonstrate that the cellulose extracted from *Myrsine andina* (Mez) Pipoly, possesses structural and chemical characteristics comparable to standard cotton cellulose, thus validating the effectiveness of the extraction method employed and the feasibility of using this cellulose in various industrial and research applications [21].

### 3.4. Optical Microscopy

#### 3.4.1. Polarized Light Optical Microscopy for Cellulose Obtained from the Species *Cedrela montana* Moritz ex Turcz

**Figure 5:**
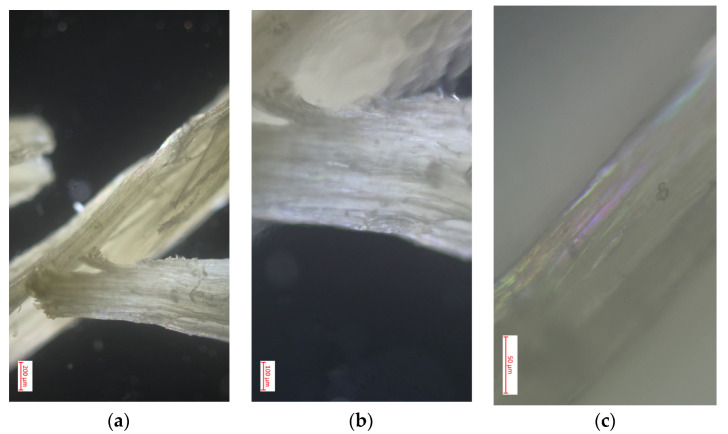
Optimal optical microscopy with polarized light performed on cellulose extracted from the species *Cedrela montana* Moritz ex Turcz: (**a**) image with 10× magnification in microscopy; (**b**) image with 40× magnification in microscopy; (**c**) image with 100× magnification in microscopy.

In Figure 5, the images obtained through polarized light optical microscopy of the cellulose from *Cedrela montana* Moritz ex Turcz reveal fascinating details of the structure of this material at different magnifications (10×, 40×, and 100×). In the first image, observed at 10×, longitudinally aligned cellulose fibers are visible, showing a clear fibrous structure and segments where the fibers intersect, reflecting the natural organization typical of cellulose in its raw state [39]. By increasing the magnification to 40×, the second image details the texture and overlapping layers of the fibers, where the distinct layers and density of the cellulose can be observed, highlighting the individual characteristics of the fibers that contribute to the material’s strength and flexibility [40]. Finally, the image at 100× shows the most detailed microscopic structure of the fibers, where the effects of polarization are evident with iridescent colors indicating a crystalline orientation in the cellulose, which is crucial for understanding the mechanical and absorption properties of cellulose. These observations demonstrate the high quality of the cellulose extracted from *Cedrela montana* Moritz ex Turcz, with a well-defined structure and molecular orientation that could be exploited in applications requiring materials with precise specifications of strength and elasticity [41].

#### 3.4.2. Polarized Light Optical Microscopy for Cellulose Obtained from the Species *Buddleja incana* Ruiz & Pav

**Figure 6:**
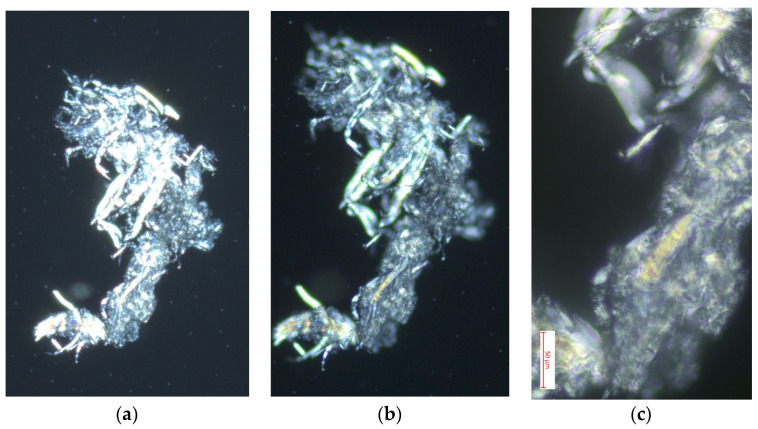
Optimal optical microscopy with polarized light performed on cellulose extracted from the species *Buddleja incana* Ruiz & Pav: (**a**) image with 10× magnification in microscopy; (**b**) image with 40× magnification in microscopy; (**c**) image with 100× magnification in microscopy.

Figure 6 shows images obtained through optical microscopy with polarized light of cellulose extracted from *Buddleja incana* Ruiz & Pav at magnifications of 10×, 40×, and 100×, revealing impressive structural details that illustrate different aspects of the organization and quality of the material. The first image, at 10×, shows cellulose fragments with an irregular and fragmented structure, where cellulose crystals exhibit a distinctive brightness under polarized light, indicative of areas with certain molecular ordering and reflecting heterogeneity in the composition and orientation of the crystals. Increasing the magnification to 40×, the second image provides a more detailed view of individual crystals, showing greater clarity in the crystalline structures and suggesting variations in the density and orientation of these [35]. The third image, at 100×, offers a close-up view of the cellulose microstructure, highlighting the reticulation patterns and fine alignment of the fibers, evidencing high crystallinity and molecular order, crucial qualities for industrial applications that require strength and durability [42]. These observations confirm the presence of well-ordered zones and less structured areas, providing valuable insights into the microscopic characteristics of the material and reflecting the quality of the extraction method used, as well as the suitability of this cellulose for specific applications where precise mechanical and chemical properties are needed [43].

#### 3.4.3. Polarized Light Optical Microscopy for Cellulose Obtained from the Species *Vallea stipularis* L. f.

**Figure 7:**
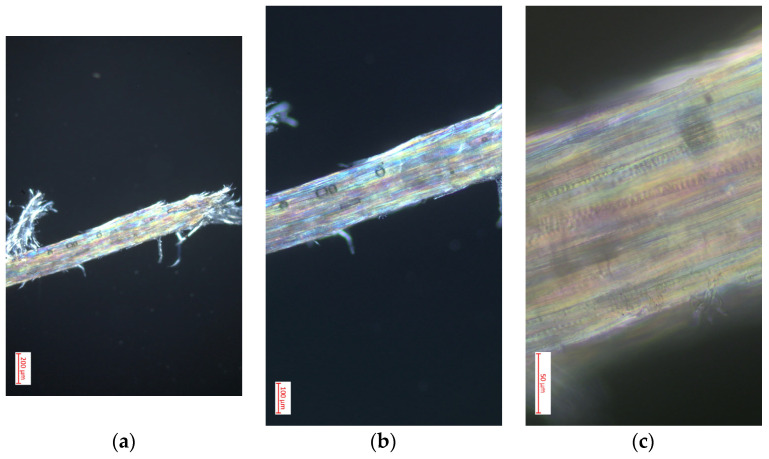
Optimal optical microscopy with polarized light performed on cellulose extracted from the species *Vallea stipularis* L. f.: (**a**) image with 10× magnification in microscopy; (**b**) image with 40× magnification in microscopy; (**c**) image with 100× magnification in microscopy.

Figure 7 presents images obtained through optical microscopy with polarized light of cellulose from *Vallea stipularis* L. f. at magnifications of 10×, 40×, and 100×, revealing distinctive structural characteristics of this material. In the 10× image, an individual fiber with frayed ends is observed, highlighting the fibrous structure and surface imperfections that are typical in natural cellulose [39]. This initial image shows how polarized light highlights the structural components, displaying different colors that indicate the orientation of cellulose crystals. As the magnification increases to 40×, the texture of the fiber is detailed more clearly, allowing observation of the layers and longitudinal alignment of cellulose, as well as areas where light is intensely dispersed, indicating variations in molecular density and alignment. The 100× image offers an even more detailed view of the internal structure of the fiber, showing the lines and patterns that are crucial for understanding the mechanical properties of cellulose [43]. This high magnification image reveals a series of bands and iridescent color patterns that evidence well-defined crystallinity and precise molecular orientation, which is essential for applications where the physical properties of cellulose are critical. These detailed observations underscore the exceptional quality of cellulose from *Vallea stipularis* L. f. and its potential for specific uses in industry where materials with detailed mechanical and absorption properties are required [40].

#### 3.4.4. Polarized Light Optical Microscopy for Cellulose Obtained from the Species *Myrsine andina* (Mez) Pipoly

**Figure 8:**
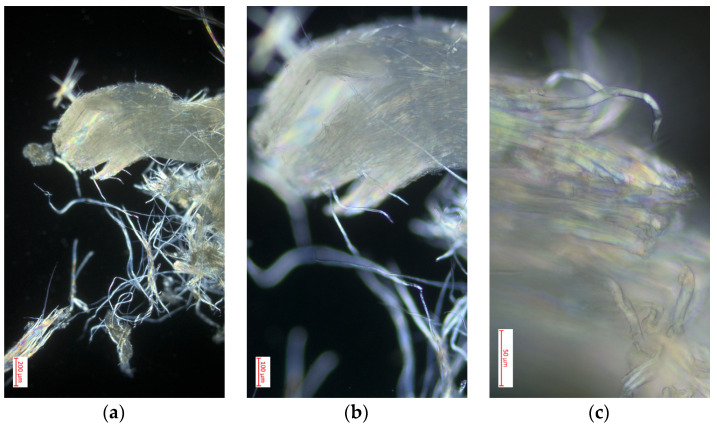
Optimal optical microscopy with polarized light performed on cellulose extracted from the species *Myrsine andina* (Mez) Pipoly: (**a**) image with 10× magnification in microscopy; (**b**) image with 40× magnification in microscopy; (**c**) image with 100× magnification in microscopy.

Figure 8 shows images obtained through optical microscopy with polarized light of cellulose extracted from *Myrsine andina* (Mez) Pipoly at magnifications of 10×, 40×, and 100×, offering a detailed view of the material’s structure. In the 10× image, a grouping of dispersed cellulosic fibers is observed with a notable crystalline brightness, characteristic of molecular orientation and the presence of highly organized regions [42]. When increasing the magnification to 40× the second image shows an individual fiber more clearly, highlighting the surfaces and edges of the cellulose, where iridescent reflections are visible, indicating the crystallinity of the material. This level of detail highlights the uniformity and precise alignment of the fibers, critical aspects for the functionality of cellulose in industrial applications [43]. Finally, the image at 100× provides a close-up of the internal structure of a fiber, where laminar layers and microfibrils are visualized. The bright colors and observed patterns are indicative of optimal molecular orientation and well-formed crystalline structure of the cellulose, which underscores its potential for uses that require specific mechanical properties and material strength. These observations confirm the high quality of cellulose extracted from *Myrsine andina* (Mez) Pipoly, revealing its intricate structure and suitability for advanced applications [41].

## 4. Discussion

The results obtained in the extraction and characterization of cellulose from forest residues of the high forest of Chimborazo, Ecuador, highlight the effectiveness of the implemented process, as well as the quality of the final product. The comparison of pulp yields, which showed statistically significant differences between the studied species, with previous studies [1,44] indicates that the methodologies used are robust and appropriate. Clauser et al. also reported success in cellulose extraction using lignocellulosic residues, which validates the methods applied for extraction and characterization [45].

Regarding the fractions of hemicellulose, lignin, ash, and wax–fat, the analyses allowed us to identify that hemicellulose represents between 24.6% and 27.8% of the raw material, suggesting a significant presence of non-cellulosic polysaccharides. Similar studies in lignocellulosic sources, such as sugarcane and corn, report hemicellulose values between 20% and 35%, which reinforces the consistency of these results with the existing literature [46,47]. Lignin, quantified by the Klason method, varied between 18.3% and 22.4%, highlighting its crucial role in the structural rigidity of the fibers. These figures coincide with studies in other species, such as pine and eucalyptus, where lignin contents vary between 15% and 30%, depending on the species and treatment method (considering that it is usual to extract cellulose from the primary trunk) [48]. The ash content, between 1.7% and 2.5%, was relatively low, indicating that the amount of inorganic components in the samples is minimal, aligning with other works on agricultural residues that report similar ash contents, such as in eucalyptus sawdust, where ash remains around 1% to 3% [49]. Finally, the wax–fat fraction, obtained through Soxhlet extraction, remained below 4.4%. This value is consistent with studies in lignocellulosic residues, where the wax–fat fraction is generally below 5%, which justifies the elimination of prior degreasing in the cellulose extraction process [50]. This simplifies the process, reducing operational costs and solvent use, making the procedure more efficient and environmentally friendly.

Biowaste from agricultural and industrial plants has been identified as a potential source of fibers for the development of biocomposite materials applicable in various industrial products. In this study, it has been demonstrated that forest residues from the high montane forest of Chimborazo can play a similar role, with high-purity cellulose fibers efficiently extracted through alkaline treatments. Compared with research on agro-residues, such as those from *Ricinus communis* (RC), where fibers treated with 4% NaOH showed higher strength and thermal stability (300–350 °C), our results highlight the viability of the obtained cellulosic fibers for applications in biocomposites [51].

Similar to the case of RC fibers, the cellulose extracted from forest residues presents a preserved crystalline structure and a high crystallinity index which, according to X-ray diffraction analyses, is in the range of 40–63%. These data support its use in the production of advanced materials. Furthermore, the textured surface of the treated fibers, observed under polarized optical microscopy and SEM, suggests that these fibers would offer good adhesion in polymeric matrices, which would increase their reinforcement capacity in biocomposites. The thermal and structural characteristics of these fibers are comparable to those observed in other biowastes, reinforcing their potential as a sustainable and economical source for the creation of green materials [52].

The statistical analysis revealed significant differences among the studied species, with *Vallea stipularis* L. f. showing the highest yield in cellulose pulp (80.83%). This difference can be attributed to variations in the composition and structure of the cell wall between species, which is consistent with previous studies that have demonstrated that the greater density and rigidity of cell walls in certain species directly influence cellulose yield [35,40]. Research conducted on other woody species, such as pine and birch, has also shown that species with thicker cell walls tend to produce higher cellulose yields due to their high concentration of structural polymers [41,53].

FTIR and polarized light microscopy analyses confirmed the successful extraction of high-purity cellulose, with spectral profiles and morphological characteristics comparable to those of standard cotton cellulose [22,54]. These results corroborate the findings [55] on similar FTIR profiles in cellulose extracted from various lignocellulosic sources [56].

The crystallinity of the samples, particularly that of *Vallea stipularis* L. f. (63.78%), falls within the range observed for other natural fibers (40–76%), as reported in several studies [57,58]. This suggests that the cellulose extracted from these Ecuadorian forest residues possesses properties comparable to those of other natural cellulose sources.

While the present study focused on alkaline treatment and the removal of hemicelluloses through simplified processes without prior degreasing, the method based on GVL and IONCELL-P achieves the removal of over 90% of hemicelluloses, reaching a purity of 96%. This high purity is also observed in our cellulose samples, especially in *Vallea stipularis* L. f., where FTIR and microscopy analyses confirmed the successful extraction of cellulose with characteristics comparable to those from traditional sources, like cotton. A key difference is that the GVL-IP method is particularly suitable for the production of high-viscosity cellulose and cellulose acetate, while the approach of the present research focuses on cellulose extraction for more general applications, such as biocomposites [59]. However, both studies highlight the viability of environmentally friendly processes for the production of high-purity cellulose and underscore the importance of optimizing extraction conditions to maximize the quality and final properties of the product without purely economic considerations, but generating a first approach to new sources of cellulose in biomass.

The extraction yields obtained (60–81%) are comparable to those reported for other biomass sources, such as eucalyptus sawdust (70%) [1]. This indicates the competitive potential of these forest residues as raw material for the production of crystalline cellulose and potential applications in nanomaterials, as discussed in the literature [60]. The importance of these findings in the context of the circular bioeconomy and sustainable development is evident, as utilizing forest residues for cellulose production contributes to the valorization of underutilized resources, which can have a significant impact on waste reduction and the promotion of a greener industry [35]. According to the analysis, these materials are not only viable for cellulose extraction but could also be employed in the creation of new composite materials, opening new avenues for research and development in the biomaterials sector [27].

## 5. Conclusions

This study on cellulose extraction from forest residues, specifically from the timber species of the high montane forest in Chimborazo, Ecuador, has demonstrated the viability and sustainability of this process. The utilization of pruning residues, including leaves, flowers, and secondary branches, has proven to be an efficient source for obtaining high-quality cellulose. This finding highlights the importance of utilizing forest by-products, thus contributing to waste reduction and enhancing resource efficiency in line with the principles of the circular bioeconomy.

The results indicate that the extracted cellulose possesses characteristics comparable to those of more traditional sources, as evidenced by the characterization and structural tests, showing high crystallinity and a molecular structure suitable for various industrial applications.

It is important to highlight that one of the main limitations of the study is that economic aspects that could support the financial viability of a biorefinery based on these residues were not addressed. This is a crucial aspect that should be evaluated in future research to validate the economic robustness of the process.

At the same time, although appropriate techniques were used for the characterization of extracted cellulose, such as FTIR and polarized light optical microscopy, it is pertinent to note that future research should incorporate more specialized analyses, such as X-ray diffraction (XRD), nuclear magnetic resonance (NMR), and scanning electron microscopy (SEM), as they can complement the results obtained.

Finally, this study establishes a solid foundation for future research aimed at optimizing cellulose extraction and processing techniques, as the proposed extraction methodology, which eliminates the need for prior degreasing, is designed to improve both the efficiency and quality of the material obtained from non-traditional residues (leaves, flowers, and secondary branches), sources of biomass that have not been studied in relation to their quantity and quality of cellulose and lignocellulosic fractions.

## Figures and Tables

**Table 1:** Mass fractions of the components in each species.

Component	*Cedrela montana* Moritz ex Turcz	*Buddleja incana* Ruiz & Pav	*Vallea stipularis* L. f.	*Myrsine andina* (Mez) Pipoly	Analytical Method
Hemicellulose (% by weight)	26.4%	27.8%	24.6%	26.9%	Hydrolysis
Lignin (% by weight)	20.1%	22.4%	18.3%	19.5%	Klason method
Ash (% by weight)	2.1%	1.7%	2.5%	2.0%	Calcination at 550 °C
Fat–wax fraction (% by weight)	2.9%	2.8%	4.4%	3.0%	Soxhlet extraction with hexane

**Table 3:** Results of the ANOVA.

	Bleached Cellulose Pulp					Cellulose Fiber				
	Df	Sum Sq	Mean Sq	F Value	*p* Value	Df	Sum Sq	Mean Sq	F Value	*p* Value
**Species**	3	584.9	194.96	22.64	0.00029 ***	3	74.03	24.68	1291	0.342
**Residuals**	8	68.9	8.61			8	152.95	19.12		

Note: Significance code: *** *p* < 0.001.

**Table 4:** Fisher LSD test.

Species	Bleached Cellulose Pulp	Groups 95% LSD = 5.52	Groups 99% LSD = 8.04
***Cedrela montana* Moritz ex Turcz**	62.96	c	b
***Buddleja incana*** Ruiz & Pav	70.45	b	b
***Vallea stipularis*** L. f.	80.83	a	a
***Myrsine andina*** (Mez) Pipoly	64.69	c	b

**Table 5:** *p*-values for the ANOVA assumption verification tests.

Assumption	Test	Cellulose Fiber (*p*-Value)	Bleached Cellulose Pulp (*p*-Value)
Normality	Shapiro–Wilk	0.1487	0.8084
Homoscedasticity	Bartlett	0.7172	0.5174
Independence	Runs	1	1

## Data Availability

The original contributions presented in the study are included in the article, further inquiries can be directed to the corresponding author.
